# Voltage-based electroanatomic mapping system for MR-guided cardiac electrophysiology: preliminary swine validations

**DOI:** 10.1186/1532-429X-15-S1-O88

**Published:** 2013-01-30

**Authors:** Zion Tse, Charles Dumoulin, Israel Byrd, Jeffrey Schweitzer, Ronald Watkins, Kim Butts Pauly, Raymond Y Kwong, Gregory F Michaud, William Stevenson, Ferenc Jolesz, Ehud J Schmidt

**Affiliations:** 1 Engineering, The University of Georgia, Athens, GA, USA; 2 Radiology, Cincinnati Children's Hospital Medical Center, Cincinnati, OH, USA; 3 Cardiovascular & Ablation Technologies, St Jude Medical Inc, St. Paul, MN, USA; 4 Radiology, Stanford University, Stanford, CA, USA; 5 Cardiology, Brigham and Women's Hospital, Boston, MA, USA; 6 Radiology, Brigham and Women's Hospital, Boston, MA, USA

## Background

MRI produces images that serve as luminal, edema, & scar maps to assist in the Electrophysiological (EP) treatment of ventricular and atrial arrhythmias [1]. Until MR-compatible EP devices are widely available, there will be a need to perform EP partially in the MRI for imaging, and partially outside the MRI for ablation, puncture & navigation. An MR-conditional voltage-based Electroanatomic Mapping (EAM) system would allow MR-guided EP in MRI & registration-free EP to be performed outside the MRI during X-ray, Intra-Cardiac-Echo (ICE) or EAM guidance. Previously a 1.5T MR-conditional St. Jude Medical EnSite Velocity (Velocity) voltage-based EAM system was presented [2]. The study objective was to conduct a multi-catheter registration free EAM (localization & intra-cardiac Electrogram (EGM) measurement) both in & outside of the MRI.

## Methods

**Figure 1:**
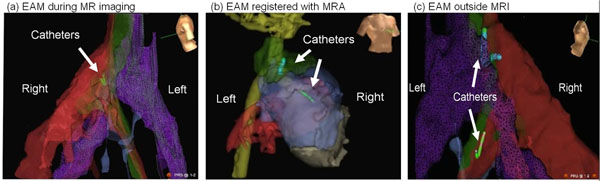
EAM allows catheter navigation inside, outside MRI and on segmented heart MRA

**Figure 2:**
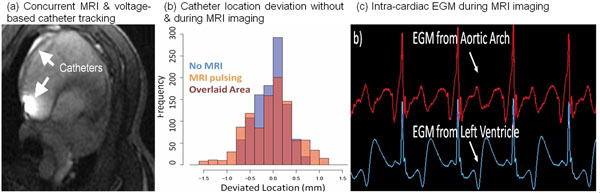
a) MRI shows catheters in the coronory sinus and the right atrium b) frequencies of deviated location of catheter electrode with no MRI (blue), with MRI pulsing (orange), and their statistically overlaid area (red). c) clean intra-cardiac EGM traces measured from catheters during MR imaging.

An MR-conditional Velocity was constructed to prevent MR gradient from reducing tracking accuracy. The system utilized an electronic switching circuit, RF-filtered electrical lines, modified surface electrode patches, & MR-conditional EP catheters [2]. Trans-septal punctures were made in 5 intubated swine under X-ray & ICE guidance. The swine were moved to a GE 1.5T MRI suite equipped with the Velocity. Prior to the procedure, 3D ECG-gated MR Angiography (MRA) provided navigational roadmaps. Three voltage-tracked EP catheters, with 4 tracked electrodes each, were navigated simultaneously inside the MRI to acquire EAM of the heart's left & right sides, with a coronary sinus catheter providing a physiological reference (Fig1). Imaging & voltage tracking were tested simultaneously (Fig2a). To measure Velocity's catheter tracking accuracy during MR imaging, catheters were navigated to specific anatomic regions, & the change in location was observed during imaging over 10-sec increments.

## Results

EAM & catheter navigation of the swine models were performed both in & outside the MRI at >20 frames-per-second without re-registration (Fig1). Imaging was conducted simultaneously with tracking (Fig2a), & catheter position remained stable during the entire imaging session (Fig1a). The median catheter electrode locations changed by 0.33-0.37 mm, while the standard deviation (SD) of the locations increased by only 0.23-0.45 mm (Fig2b). Since some of the positional SD was due to respiratory or cardiac motion, this slight increase in positional oscillation was hard to visually detect in EAM. Concurrent imaging & tracking were successful during sequences with TR>32 ms, capturing cardiac tissue during critical procedural stages. Image quality reduction of <5% was shown in FSE & GRE sequences. High-fidelity Intra-cardiac EGMs were obtained even during imaging (Fig2c). Electrode heating was <1oC under sequences of 4 Watt/kg.

## Conclusions

MRI-conditional voltage tracking allows simultaneous catheter tracking & MR imaging, permitting registration-free EAM in& outside MRI during EP procedures.

## Funding

NIH U41-RR019703, R43 HL110427-01, AHA 10SDG261039.
